# Supplementary material for: Development of a computer-aided design software for the quantitative evaluation of aesthetic damage
Source: PLoS One. 2019 Dec 18;14(12):e0226322. doi: 10.1371/journal.pone.0226322 (PMC6919621; doi:10.1371/journal.pone.0226322)
Supplement: S1 Code site — 1. (ZIP) [file pone.0226322.s001.zip › [EESC_jr][Projeto]Relatorio_07_21/vendors/select2/docs/_includes/examples/basics.html]

# The basics

## Single select boxes

Select2 can take a regular select box like this...

and turn it into this...

{% highlight html linenos %}
Alabama
...
Wyoming
{% endhighlight %}

## Multiple select boxes

Select2 also supports multi-value select boxes. The select below is declared with the `multiple` attribute.

{% highlight html linenos %}
Alabama
...
Wyoming
{% endhighlight %}

## Select boxes with labels

You can, and should, use a `<label>` with Select2, just like any other `<select>` element.

Click this to highlight the single select element

Click this to highlight the multiple select element

{% highlight html linenos %}
Click this to highlight the single select element

Click this to highlight the multiple select element
{% endhighlight %}
